# Multifaceted roles of transcription factors during plant embryogenesis

**DOI:** 10.3389/fpls.2023.1322728

**Published:** 2024-01-03

**Authors:** Hai Ying Yuan, Sateesh Kagale, Alison M. R. Ferrie

**Affiliations:** Aquatic and Crop Resource Development Research Center, National Research Council Canada, Saskatoon, SK, Canada

**Keywords:** AP2/ERF, B3, homeobox, somatic embryogenesis, transcription factor, zygotic embryogenesis

## Abstract

Transcription factors (TFs) are diverse groups of regulatory proteins. Through their specific binding domains, TFs bind to their target genes and regulate their expression, therefore TFs play important roles in various growth and developmental processes. Plant embryogenesis is a highly regulated and intricate process during which embryos arise from various sources and undergo development; it can be further divided into zygotic embryogenesis (ZE) and somatic embryogenesis (SE). TFs play a crucial role in the process of plant embryogenesis with a number of them acting as master regulators in both ZE and SE. In this review, we focus on the master TFs involved in embryogenesis such as BABY BOOM (BBM) from the APETALA2_/_Ethylene-Responsive Factor (AP2/ERF) family, WUSCHEL and WUSCHEL-related homeobox (WOX) from the homeobox family, LEAFY COTYLEDON 2 (LEC2) from the B3 family, AGAMOUS-Like 15 (AGL15) from the MADS family and LEAFY COTYLEDON 1 (LEC1) from the Nuclear Factor Y (NF-Y) family. We aim to present the recent progress pertaining to the diverse roles these master TFs play in both ZE and SE in Arabidopsis, as well as other plant species including crops. We also discuss future perspectives in this context.

## 1 Overview of transcription factors

Transcription factors (TFs) are regulatory proteins that participate in the regulation of gene transcription. TFs have DNA-binding domains that bind to specific DNA regulatory sequences. TFs exhibit dual capabilities depending on their binding sites: they can facilitate transcription initiation when binding to DNA promoter sequences, or alternatively, they can activate or repress gene transcription when binding to enhancer sequences (Wang et al., 2015). TFs can be ubiquitous and exist in all cell types, or they can be specialized and only exist in certain cell types or certain developmental stages. There are a large number of transcription factors in living organisms including plants. Arabidopsis has close to 2300 TFs based on the recent classification, which corresponds to ~8.3% of its total genes (Hong, 2016). Crop species have a similar percentage of TFs in their genomes with 5.7% in wheat (*Triticum aestivum*), 6.5% in rice (*Oryza sativa*), and 6.1% in canola (*Brassica napus*) (Priya and Jain, 2013; Zheng et al., 2016; Evans et al., 2022). Fruit fly (*Drosophila melanogaster*) has a similar genome size as Arabidopsis, but the recent classification has revealed it has only ~5.5% TFs (Pfreundt et al., 2010; Shokri et al., 2019). Similarly, mouse (*Mus musculus*) and maize (*Zea mays*) have comparable genome sizes, while the former has ~6.8% TFs as compared to ~8.3% in maize (Jin et al., 2017; Zhou et al., 2017). The abundance, variety, and remarkable diversity of DNA-binding specificities exhibited by plant TFs, when compared to their counterparts in animals with similar genome sizes, suggest a potentially more important role for TFs and their transcriptional regulations in plants (Shiu et al., 2005; Mitsuda and Ohme-Takagi, 2009).

As the initial step in governing gene expression, transcriptional regulation has a direct impact on proteome, metabolome, and phenome. Because of the diverse roles of TFs, cells possessing the same genome within an organism can have different functions. TFs have been shown to play important roles in various growth and developmental processes. Morphogenesis-related processes such as light-controlled seedling morphology, the formation of floral traits, fruit morphology, as well as thermomorphogenesis, where the morphology changes under high temperatures, all have TFs’ involvement (Sasaki, 2018; Shi et al., 2018; Han et al., 2019; Zhang et al., 2020a; Chopy et al., 2023). TFs also modulate organogenesis such as leaf, shoot and root development, or even nodule development in the symbiotic relationship between legume and rhizobium (Sluis and Hake, 2015; Tu et al., 2021; Chakraborty et al., 2022; Liu et al., 2022). Gene transcriptional regulation, facilitated by TFs, is essential in controlling numerous biological processes in plants, including signal transduction, stress and defense responses, as well as carbohydrate metabolism (Seo and Choi, 2015; Hoang et al., 2017; Shahzad et al., 2021; Strader et al., 2022; Zou and Sun, 2023).

## 2 Plant embryogenesis

Embryogenesis is a process where embryos form and develop. Plant embryogenesis starts from non-embryogenic cells. These non-embryogenic cells can be unfertilized egg cells for most flowering plants including crop species, but they can also be any cells that eventually develop into embryo-like structures and are capable of further developing into plants (de Vries and Weijers, 2017). The process of fertilizing egg cells to form zygotes and further develop into embryos under natural conditions is usually referred to as zygotic embryogenesis (ZE), while the process of embryos developing from any other cells without fertilization is usually referred to as somatic embryogenesis (SE). SE can occur naturally within an organism, as seen in cases like apomixis where embryos develop *in vivo* from unfertilized ovules, and parthenogenesis, where embryos develop from unfertilized egg cells. It is also not uncommon in plants that cells of different origins such as somatic cells or even microspores develop into embryos under inducive *in vitro* culture conditions.

**Figure 1:**
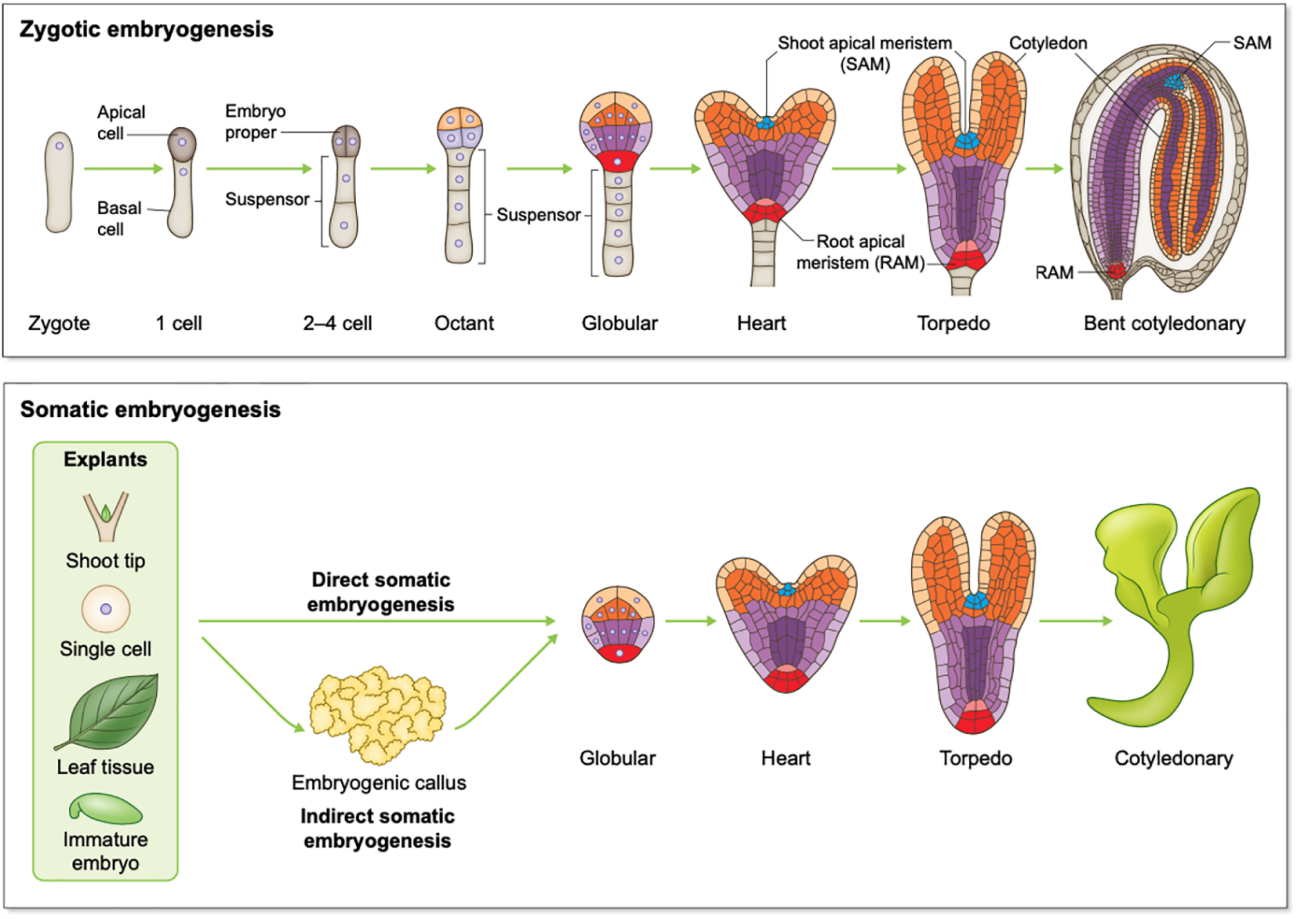
Schematic overview of plant embryogenesis. Top panel: Arabidopsis zygotic embryogenesis from the zygote to the bent cotyledonary stage; Bottom panel: somatic embryogenesis in dicots.

Embryogenesis is a multi-stage process irrespective of whether it is ZE or SE. ZE involves a zygote developing through stages including 1-cell, 2-cell to octant, globular, heart, and finally a mature cotyledonary embryo for dicotyledon species (dicots) such as Arabidopsis (**Figure 1**). There are classic reviews regarding Arabidopsis ZE that readers can refer to including Capron et al., Wendrich et al., and ten Hove et al. (Capron et al., 2009; Wendrich and Weijers, 2013; Ten Hove et al., 2015). Zygotic embryos developed from monocots such as cereal crops wheat, rice and maize are morphologically different from those developed in dicots. However, developmental processes such as pattern formation and transcriptional regulation of ZE are conserved to a remarkable extent between monocots and dicots (Nardmann et al., 2007; Zhao et al., 2017). For readers interested in ZE in monocot species, we recommend the following comprehensive reviews of Vernoud et al., and Kruglova et al.(Vernoud et al., 2005; Kruglova et al., 2022). Despite originating differently, SE shares high similarities with ZE at morphological, physiological and molecular levels, and both processes share developmental stages like globular, heart, torpedo, and cotyledonary stages (Ikeda et al., 2006; Winkelmann, 2016). During SE, sometimes an intermediate stage involving embryogenic callus occurs, and this process is referred to as indirect SE (**Figure 1**). For readers interested in SE, the following comprehensive reviews of Smertenko and Winkelmann can be a good starting point (Smertenko and Bozhkov, 2014; Winkelmann, 2016).

Whether it is ZE or SE, the diverse origin of plant embryos signifies substantial changes occurring during embryogenesis. The acquirement of embryogenic competence and continued development requires the regulation and coordination of a myriad of genes, various gene networks and factors. Epigenetic control such as chromosome remodeling, transcriptional gene regulation and hormonal regulation have been associated with plant embryogenesis (Gulzar et al., 2020; Armenta-Medina et al., 2021); however, there is still a need for more work to fully understand the molecular mechanisms involved in plant embryogenesis. Such insights could open doors to opportunities like engineering crop embryos with desired traits/characteristics.

## 3 Transcription factors in plant embryogenesis

TFs play key roles during plant embryogenesis. From cell fate determination and apical-basal patterning initiation to embryonic shoot, and root formation, various members of different TF families have been shown to be essential for these processes (Le et al., 2010; Horstman et al., 2017a; Méndez-Hernández et al., 2019; Gulzar et al., 2020; Gundu et al., 2020; Tian et al., 2020; Verma et al., 2022). TFs such as BABY BOOM (BBM) from the APETALA2_/_Ethylene-Responsive Factor (AP2/ERF) family, WUSCHEL and WUSCHEL-related homeobox (WOX) from the homeobox family, LEAFY COTYLEDON 2 (LEC2) from the B3 family are some of the master regulators that perform essential roles during plant embryogenesis. In this concise review, we present the latest developments in understanding the varied roles and functions of these master TFs in plant embryogenesis, along with a list of TFs that are capable of inducing somatic embryogenesis in different plant species (**Table 1**).

**Table 1:** Transcription factors used to induce somatic embryogenesis in plants.

Transcription Factor	TF family	Plant Species	Approach	References
AGL15	MADS family	*Arabidopsis thaliana, Glycine max*	Genetic transformation	Thakare et al., 2008; Zheng and Perry, 2014,
AGL18	MADS family	*Glycine max*	Genetic transformation	Zheng and Perry, 2014,
BBM	AP2/ERF family	*Arabidopsis thaliana, Brassica napus, Theobroma cacao, Capsicum annuum, Populus tomentosa, Nicotiana tabacum, Sorghum bicolor, Zea mays, Malus domestica, Oryza sativa, Gossypium hirsutum, Solanum Lycopersicon*	Genetic transformation, Gene editing	Boutilier et al., 2002; Srinivasan et al., 2007; Deng et al., 2009; Heidmann et al., 2011; Florez et al., 2015; Mookkan et al., 2017; Mookkan et al., 2017; Yavuz et al., 2020; Nelson-Vasilchik et al., 2022; Qi et al., 2022; Khanday et al., 2023; Xiao et al., 2023, Chen et al., 2022a
BBM/WUS2	AP2/ERF family/Homeobox family	*Zea mays, Sorghum bicolor, Eragrostis tef, Panicum virgatum, Cenchrus americanus, Setaria italica, Triticum aestivum, Secale cereale, Hordeum vulgare, Saccharum officinarum, Oryza sativa*	Genetic transformation, gene editing	Lowe et al., 2018; Aregawi et al., 2020; Hoerster et al., 2020; Peterson et al., 2021; Aregawi et al., 2022; Beyene et al., 2022, Xu et al., 2022b, Nelson-Vasilchik et al., 2022; Johnson et al., 2023; Wang et al., 2023,
FUS3	B3 family	*Citrus unshiu, Arabidopsis thaliana*	Genetic transformation	Liu et al., 2018,
GRF	GRF family	*Zea mays*	Genetic transformation	Kong et al., 2020,
GRF-GIF	GRF family/GIF family	*Triticum aestivum*	Genetic transformation, gene editing	Debernardi et al., 2020,
LEC1	NF-Y family	*Arabidopsis thaliana*, *Nicotiana tabacum, Manihot esculenta, Oryza sativa*	Genetic transformation, gene editing	Guo et al., 2013; Pelletier et al., 2017; Brand et al., 2019; Niu et al., 2021,
LEC2	B3 family	*Nicotiana tabacum, Manihot esculenta, Theobroma cacao*	Genetic transformation	Guo et al., 2013; Shires et al., 2017; Brand et al., 2019; Li et al., 2019,
RKD	RKD family	*Arabidopsis thaliana, Oryza sativa, Citrus sinensis*	Genetic transformation	Waki et al., 2011; Shimada et al., 2018; Purwestri et al., 2023,
WOX9	Homeobox family	*Medicago truncatula*	Genetic transformation	Tvorogova et al., 2019,
WOX2a	Homeobox family	*Zea mays*	Genetic transformation	Mcfarland et al., 2023,
WUS	Homeobox family	*Coffea canephora, Gossypium hirsutum, Medicago truncatula, Zea mays*	Genetic transformation	Arroyo-Herrera et al., 2008; Bouchabké-Coussa et al., 2013; Mookkan et al., 2017; Kadri et al., 2021,

### 3.1 AP2/ERF family

The AP2/ERF family is one of the largest TF families in plants and plays an essential role in development processes and stress responses. Members within the AP2/ERF family share a common DNA-binding domain – AP2 domain and they are further categorized into 4 sub-families based on the copy number difference and the sequence variation of the domain (Gu et al., 2017). Within the AP2/ERF family, BABY BOOM/PLETHORA4 (BBM/PLT4) is the key player in plant embryogenesis. In addition, BBM-like genes including other PLT genes are increasingly recognized as important players in plant embryogenesis from recent studies.

The expression of Arabidopsis BBM is detected not only in the embryo starting as early as at the zygote stage, but also in the chalazal region of the ovule and the endosperm cells at the early initiating stage, suggesting a broader range of functions for BBM in both embryo and endosperm development in Arabidopsis (Chen et al., 2022a). BBM gene in Arabidopsis didn’t show parent-of-origin expression patterns during embryo development and the analyses of single, and double mutants of BBM and PLT2 created using CRISPR-Cas9 showed that both genes coordinately work together in maintaining embryo development beyond the 4-cell stage, as well as regulating cell division planes and cell shapes (Chen et al., 2022a). Maize (*Zea mays*) BBM-like genes *ZmBBML1* and *ZmBBML2* showed induced expression 12 hours after pollination with the increased expression of *ZmBBML3* at 24 hours after pollination and after zygote division (Chen et al., 2017). The expression of rice BBM gene *OsBBM1* was highly induced during the initiation of embryogenesis (Anderson et al., 2017). In addition, rice BBM1 from the male parent initiated embryo development in the fertilized egg cell and subsequently stimulated BBM1 from the female parent to work together on embryo patterning, which is different from Arabidopsis (Khanday et al., 2018; Chen et al., 2022a). Wheat BBM homologs *TaBBM-gA* and *TaBBM-gD* were also induced in an embryogenic microspore population (Bilichak et al., 2018).

Earlier studies have shown that BBM overexpression induces somatic embryo development in many species including *Arabidopsis thaliana*, canola (*Brassica napus*), cocoa (*Theobroma cacao*), sweet pepper (*Capsicum annuum*), poplar (*Populus tomentosa*), and tobacco (*Nicotiana tabacum*) as shown in **Table 1** (Boutilier et al., 2002; Srinivasan et al., 2007; Deng et al., 2009; Heidmann et al., 2011; Florez et al., 2015). Overexpression of rice *OsBBM1* in the egg cell has been demonstrated to induce parthenogenesis, an embryo development process without fertilization. In contrast, the generation of triple knock-out mutants involving rice BBM1, BBM2, and BBM3 through gene editing resulted in the abortion of developing embryos during the early stage at 5 days after pollination (Khanday et al., 2018). CRISPR activation system targeting maize BBM2 (*ZmBBM2*) in egg cells also induced parthenogenesis (Qi et al., 2022). In a similar study, ectopic expression of *B. napus* BBM gene *BnBBM1* in the egg cells of Arabidopsis, canola, and tomato was able to induce haploid embryos in all three species (Chen et al., 2022a). *PsASGR-BABY BOOM-like* (*PsASGR-BBML*) gene, a member of the BBM-like AP2/ERF transcription factors, originates from an apomictic species of pearl millet, *Pennisetum squamulatum* (Conner et al., 2015). When introduced as a transgene, the *PsASGR-BBML* has been shown to induce parthenogenesis in sexual tetraploid pearl millet, maize, rice, and even tobacco, a dicot species (Conner et al., 2015; Conner et al., 2017; Zhang et al., 2020b). Most recently, overexpression of any of the three BBM genes from foxtail millet (*Setaria italica*): *SiBBM1, SiBBM2* and *SiBBM3* in egg cells induced parthenogenesis in rice (Chahal et al., 2022). Overexpression of *MdBBM* promoted somatic embryogenesis in cultured young leaves of apple (*Malus domestica*) (Xiao et al., 2023). This further confirms the conservation of BBM functions among monocot and dicot species.

Elevated expression of BBM-like PLT gene members, such as *PLT1, PLT2, PLT3*, and *PLT7*, has been demonstrated to induce somatic embryogenesis. Notably, *PLT1* and *PLT3* were found to recover early-stage embryo defects of *plt2 bbm* mutants, suggesting a redundant and overlapping role of BBM and BBM-like genes in embryogenesis (Horstman et al., 2017b; Kerstens et al., 2022). In addition, BBM and PLT2-induced somatic embryogenesis is dosage-dependent with higher levels of BBM/PLT2 resulting in the initiation of embryogenesis (Horstman et al., 2017b). This also aligns with previous research indicating that transcription factors have maintained a dosage-dependent pattern following historical polyploidization events (Birchler and Veitia, 2007). The broad applications and evidence of BBM and BBM-like genes in both zygotic and somatic embryogenesis show that they play important roles in plant embryogenesis.

### 3.2 Homeobox family

The homeobox family is a large family of TFs with a DNA-binding domain called homeodomain (HD) and plays important roles in various development processes including organism differentiation as well as increasing developmental complexity (Mukherjee et al., 2009; Lutova et al., 2015; Jha et al., 2020). Within the family, WUSCHEL (WUS) and WUSCHEL-LIKE HOMEOBOX (WOX) are key players in plant embryogenesis.

WUS has been reported to regulate stem cell fate in Arabidopsis as early as the late twentieth century (Mayer et al., 1998). Similarly, studies have shown that after zygote division, embryo development is directed by the apical-cell-expressed WOX2, while basal-cell-expressed WOX8 governs suspensor development and root initiation, as well as regulating WOX2 expression during early embryogenesis (Breuninger et al., 2008). The enrichment of WOX8/WOX9 in the basal cells was further confirmed from Arabidopsis early embryos at the single-cell level (Kao et al., 2021). Members of the WOX family play specific roles during embryogenesis such as embryonic development, preservation of meristematic stem cells, formation of lateral organs, seed production, and regeneration of separated tissues and organs (Zhang et al., 2016; Jha et al., 2020). Recent work shows that WOX8 expression is further controlled by both paternal and maternal regulators (Ueda et al., 2017). As a master regulator of embryogenesis, WOX8 integrates the signals from both maternal and paternal factors to regulate embryo patterning through the initiation of the asymmetric division of the zygote.

The expression patterns of WUS and WOX5 are conserved in shoot and root apical meristem during early embryogenesis as demonstrated through the analysis of 13 WOX family members from the tobacco (*Nicotiana tabacum*) genome as compared to their counterparts in Arabidopsis (Zhou et al., 2018). The stage-specific expression pattern was identified in tobacco with the expression of five WOXs (WOX2, WOX9, WOX11, WOX13a, and WOX13b) started as early as 2-cell proembryo stage, while WUS and WOX5 only started at 8-cell embryo stage. In addition, the analysis revealed that WOX genes in tobacco displayed parent-of-origin effects, and the formation of embryo pattern is established post-fertilization involving the expression of WOX2 and WOX9 in the zygote, which differs from the situation in Arabidopsis. However, cell-type specific expression patterns of WOXs in the apical/basal cells were conserved between maize, tobacco and Arabidopsis (Chen et al., 2017; Zhou et al., 2018). In addition, WOX8/9 was identified as the most prevalent suspensor-specific TF at globular embryos and had a similar role in suspensor development in scarlet runner bean (*Phaseolus coccineus*), common bean (*Phaseolus vulgaris*), and soybean (*Glycine max*), similar to Arabidopsis (Chen et al., 2021). The rice orthologs of Arabidopsis WOX8/9 and WOX2 show increased expression at 2.5 and 5 hours after pollination, indicating their roles during the early stage of embryogenesis (Anderson et al., 2017). In addition, the parent of origin for WOX8/9 expression was solely paternal (Anderson et al., 2017). Similarly, Maize WOX genes *ZmWOX9A* and *ZmWOX9B*, homologs of Arabidopsis WOX8 and WOX9, showed increased expression at 12 hours after pollination and had higher expression in basal cells as in Arabidopsis (Chen et al., 2017).

Subsequently, it has been shown that embryogenic stem cell regeneration relies on WUS during somatic embryogenesis in Arabidopsis (Su et al., 2009). WUS expression was upregulated in embryogenic calli before somatic embryos developed. *HvWUS* exhibits higher expression when immature embryos are used as the explants compared to mature embryos, thus facilitating the induction of embryogenic callus formation in barley (*Hordeum vulgare*) (Suo et al., 2021). Overexpression of the Arabidopsis WUS gene promotes somatic embryogenesis in various plant species including coffee (*Coffea canephora*), cotton (*Gossypium hirsutum*), and barrel clover (*Medicago truncatula*) (Arroyo-Herrera et al., 2008; Bouchabké-Coussa et al., 2013; Kadri et al., 2021). The inclusion of the WUS gene within the gene transformation cassette promotes somatic embryogenesis of both leaf and hairy root explants in barrel clover without the need for plant growth regulators. Additionally, it enables the transformation of the historically recalcitrant maize and sorghum (*Sorghum bicolor*) genotypes using immature embryos as explants (Mookkan et al., 2017; Aregawi et al., 2020; Hoerster et al., 2020; Kadri et al., 2021). Furthermore, when under the control of tissue-specific, and auxin-inducible promoters, the BBM/WUS2 transgene triggers swift and direct somatic embryogenesis in maize in a genotype-independent manner (Lowe et al., 2018). The co-expression of maize BBM and WUS genes also improves somatic embryogenesis in recalcitrant maize and sorghum genotypes (Mookkan et al., 2017; Nelson-Vasilchik et al., 2022).

*LdWOX2*, a homolog of Arabidopsis WOX2 in European larch (*Larix decidua*), was highly expressed during early embryogenesis in somatic embryos (Rupps et al., 2016). Three different barrel clover lines were used to test the effects of overexpression of *MtWOX9-1* using leaves as the explants and the results showed *MtWOX9-1* promoted somatic embryogenesis as well as led to the expression changes of two embryogenesis-associated MADS-box genes (Tvorogova et al., 2019). Similarly, WOX2a promotes somatic embryogenesis in recalcitrant maize genotypes (Mcfarland et al., 2023). Overexpression of the WOX2a gene from an embryogenic maize genotype A188 produced somatic embryos from a recalcitrant genotype B73. In addition, the overexpression of the WOX2a gene from B73 had a similar effect on somatic embryo production. The key roles of both WUS and WOXs in somatic embryogenesis underscores their significant contributions to plant embryogenesis.

### 3.3 B3 family

The B3 family is among the most extensive plant-specific transcription factor families. Its members may feature a single B3 domain as in ARF (AUXIN RESPONSE FACTOR) and LAV (LEAFY COTYLEDON2-ABSCISIC ACID INSENSITIVE3-VAL) subfamilies, or they can possess as many as six B3 domains as observed in REM (REPRODUCTIVE MERISTEM) subfamily (Swaminathan et al., 2008). RAV (RELATED TO ABI3/VP1) subfamily is unique in that some members within the subfamily contain both a B3 domain and an AP2 domain, and the binding sites need to have sequences for both domains.

Members within the LAV subfamily of B3 transcription factors such as ABA INSENSITIVE3 (ABI3), FUSCA3 (FUS3), LEAFY COTYLEDON 2 (LEC2), VIVIPAROUS1/ABI3-LIKE1(VAL1), VAL2 and VAL3 function as regulators of embryogenesis and are part of the embryogenesis-related genes in Arabidopsis (Swaminathan et al., 2008; Carbonero et al., 2017; Kumar et al., 2020). An earlier investigation has demonstrated that Arabidopsis LEC2 is involved in embryonic cell fate maintenance during early embryogenesis, as well as the initiation of embryo maturation during the late embryogenesis stage (Stone et al., 2008). Within the LAV subfamily, AFL (ABI3/FUS3/LEC2) plays an important role in embryogenesis and the studies on the effects of individual AFL loss-of-function mutants revealed the redundancy of their function on the establishment of embryo morphology, more specifically, the cotyledon shape and bending (Devic and Roscoe, 2016). Spatiotemporal regulation of FUS3 expression in ovule integuments and endosperm ensures a coordinated embryo and endosperm growth (Wu et al., 2020). Through a single-nucleus RNA-seq study of Arabidopsis early embryos, FUS3 was shown to be highly expressed in the basal cell controlling suspensor development and root initiation among other TFs (Kao et al., 2021).

Five AFL orthologs were identified in maize with *ZmAFL2*, a FUS ortholog, *ZmAFL3/ZmVP1*, an ABI3 ortholog and *ZmAFL4, ZmAFL5, ZmAFL6*, orthologs of LEC2 (Grimault et al., 2015). *ZmAFL* genes consecutively expressed at different stages, with the peak expression of *ZmAFL2, ZmAFL5*, and *ZmAFL6* as early as 3 days after pollination (DAP), while *ZmVP1* reached peak expression at 35 DAP during zygotic embryogenesis. Maize AFLs also showed distinct spatial expression patterns with *ZmAFL2* and *ZmVP1* specifically expressed in the embryos, while *ZmAFL4* was mostly expressed in the endosperm. Though there is a difference in spatial gene expression of AFL genes between Arabidopsis and Maize, the sequential expression of FUS3 and ABI3 is maintained. Barley ABI3 ortholog *HvVP1* expressed in both embryos and endosperms and showed higher expression at the immature embryo stage (20 DAP) (Abraham et al., 2016). Both *MtFUS3-like* and *MtABI3-like* showed the highest expression at the torpedo stage embryos during zygotic embryogenesis in barrel clover, and *MtABI3-like* expressed throughout the embryo proper, but not in the suspensor (Kurdyukov et al., 2014).

As for Arabidopsis embryogenesis *in vitro*, FUS3 and ABI3 were significantly upregulated in early-stage somatic embryos (Day 5 and Day 10 after the induction), and ABI3, FUS3 and LEC2 were highly expressed in somatic embryos as compared to leaf tissues in a global scale transcriptomic study (Wickramasuriya and Dunwell, 2015). In addition, ectopic expression of LEC2 induces somatic embryogenesis and the LEC2-overexpressing explants show higher somatic embryo induction under low auxin concentration (Wójcikowska et al., 2013). Three AFL genes from barrel clover *MtLEC2, MtFUS3*, and *MtABI3* show higher expression 10 days after initiating the culture in an embryogenic genotype as compared to no or low expression in a non-embryogenic genotype (Barreto et al., 2019). *CaABI3* is highly expressed in embryogenic cells/calli and could be used as a biomarker for somatic embryogenesis in coffee, while *CaVAL2* shows higher expression in cotyledonary embryos, a later stage in embryogenesis (Freitas et al., 2019). In addition, FUS3 is expressed all through somatic embryogenesis in coffee with the highest expression in globular embryos (Awada et al., 2023). Overexpression of the *CsFUS3* gene from sweet orange (*Citrus sinensis*) promotes somatic embryogenesis in recalcitrant Satsuma mandarin (*Citrus unshiu*) and restores embryogenesis in Arabidopsis *fus3* mutants (Liu et al., 2018). In addition, three other B3 TFs (*CsABI3, CsABI5*, and *CsVAL1*) show significantly higher expression in the *CsFUS3-*overexpression lines as compared to the control in sweet orange. Overexpression of LEC2 ortholog induced somatic embryogenesis from leaf explants in cocoa (Shires et al., 2017), while a single *MeLEC2* overexpression was able to induce somatic embryogenesis in cassava (*Manihot esculenta*) (Brand et al., 2019). Though copy numbers of AFL genes vary among monocot and dicot species, their significance in plant embryogenesis remains consistent.

### 3.4 MADS family

The MADS transcription factor family is an ancient group of transcription factors with a considerably expanded number of family members in plants, which share a core DNA-binding domain: MADS domain (Gramzow and Theissen, 2010). Initially identified as a major factor in controlling flower development, the MADS TF family has been shown to play important roles in various developmental processes including pollen and embryo sac development, seed development and fruit development (Theißen and Gramzow, 2016). Within MADS TFs, AGAMOUS-Like 15 (AGL15) and AGL18 are two key members in plant embryogenesis.

Previous immunohistochemical studies have shown that AGL15 is highly expressed in embryogenic tissues derived from various sources, including Arabidopsis and maize zygotic embryos, dandelion (*Taraxacum officinale*) apomixis embryos, canola microspore-developed embryos, as well as alfalfa (*Medicago sativa*) somatic embryos (Perry et al., 1999). This indicates a much-conserved role for AGL15 in embryo development across plant species. AGL15 has been shown to directly target AFL (ABI3/FUS3/LEC2) genes, the key regulators of embryogenesis, through a genome-wide binding-site identification study (Zheng et al., 2009). Among the MADS genes, 23 were specifically expressed in embryogenic tissues of Arabidopsis, with several of them displaying differential expression across various stages of somatic embryo development (Wickramasuriya and Dunwell, 2015). Among these genes, AGL15 exhibited higher expression levels in Arabidopsis somatic embryos compared to leaf tissues. Mutations in the rice MADS gene FEMALE-STERILE (FST) lead to a complete embryo abortion (Lee et al., 2013). Analyses of gene expression changes in *fst* mutants show that genes involved in auxin transportation, cell differentiation, and embryogenic development were down-regulated.

Arabidopsis AGL18 and AGL15 have redundant functions in promoting somatic embryogenesis (Paul et al., 2022). They interact in somatic embryo tissues and the phosphorylation of both AGL15 and AGL18 is pivotal to the process through combined chromatin immunoprecipitation (ChIP-seq) and RNA-seq studies. In addition, AGL15 transgenic seedlings showed increased somatic embryo induction at 64.4%, and AGL18 transgenic seedlings had a 40.8% induction rate as compared to a 19.8% induction rate in wild-type seedlings. In a more recent study, MADS gene AGL62 was identified as the gene responsible for activating small invertase inhibitors in the syncytial endosperm and controlling the rate of embryo growth in Arabidopsis (Hoffmann et al., 2022).

*MtAGL15* expression increased in *MtWOX9-1* overexpressing calli which showed increased somatic embryogenesis capacity (Tvorogova et al., 2019). Coffee AGL15 is expressed during the early stages of somatic embryogenesis, albeit at a relatively modest level (Awada et al., 2023). An earlier study shows that overexpression of Arabidopsis AGL15 promotes somatic embryo development in both Arabidopsis and soybean (Thakare et al., 2008). Constitutive expression of soybean *GmAGL15* or *GmAGL18* sped up and increased somatic embryogenesis in soybeans, with ABI3 and FUS3 directly upregulated by *GmAGL15* during the process (Zheng and Perry, 2014). Three AGL15 homologs *GhAGL15-1, GhAGL15-3* and *GhAGL15-4* were isolated from cotton and their expressions were found to be increased during the somatic embryogenesis process (Yang et al., 2014). Overexpression of any of the three *GhAGL15s* in hypocotyls dramatically increased embryogenic callus formation, with *GhAGL15-4* having the highest increase in formation rate from 38.1 to 65.2%. The AGAMOUS subfamily of MADS TFs has long been shown to be a key player in embryogenesis, however, there is a lack of recent progress, particularly in understanding the functions of their corresponding homologs in monocot species.

### 3.5 NF-Y family

The Nuclear Factor Y (NF-Y) family is yet another transcription factor family that has much-expanded family members in plants. NF-Y TFs are also called Heme-associated proteins (HAPs) or CCAAT box binding factors (CBFs) and they form a heterotrimeric complex with one single subunit each of NF-YA, NF-YB and NF-YC to bind at CCAAT sites to regulate various developmental processes (Petroni et al., 2012). Initial research has indicated the significance of LEAFY COTYLEDON gene (LEC1, NF-YB9) in both early and late embryo development regulating processes such as embryonic cell fate, cotyledon identity and embryo maturation (Lotan et al., 1998). However, a growing body of evidence suggests that additional members within the family including LEC1– like (LIL, NF-YB6) also play crucial roles (Kwong et al., 2003; Fornari et al., 2013; Mu et al., 2013).

Eight of ten Arabidopsis NF-YA genes show expression in embryos (Siriwardana et al., 2014). Arabidopsis NF-YA3 and NF-YA8 are functionally redundant and are required in early embryogenesis (Fornari et al., 2013). *In situ* hybridization showed that both NF-YA3 and NF-YA8 have the highest expression during the early embryo development stages. Embryos from *nf-ya3 nf-ya8* double mutants or RNAi suppression exhibit defects and do not progress to the heart stage. Arabidopsis NF-YA1, 5, 6, and 9 have redundant roles in multiple developmental processes including male gametophyte development, embryogenesis, and seed development (Mu et al., 2013). NF-YA1, 5, 6, and 9 co-express with LEC1 in similar developmental windows from the early heart stage to the late torpedo stage and nf-ya1 mutants show embryo development defects at the early heart stage. Among the ten Arabidopsis NF-YA genes, NF-YA1 and NF-YA9 are most similar, as well as NF-YA5 to NF-YA6. The similarity of functions among LEC1 and LIL (NF-YB subunit), and NF-YA1, 5, 6, and 9 suggests they may be part of the NF-Y heterotrimeric complex involved in the regulation of embryogenesis. A recent study demonstrated that Arabidopsis LIL/NF-YC3/NF-YA6 trimers specifically bind to the CCAAT motif, but not the separate subunit (Gnesutta et al., 2017). This further shows that LEC1 and LIL, both NF-YB subunits, need to form a heterotrimeric complex with the other two NF-Y subunits (NF-YA and NF-YC) to function properly.

RNA-seq study of developing seeds from Arabidopsis *lec1-1* mutant shows that genes affected by *lec1-1* mutation are mostly expressed in embryo and endosperm, and expressions of TFs such as BBM, WOX2, FUS3, and ABI3 were down-regulated in *lec1-1* mutants (Pelletier et al., 2017). Soybean has four LEC1 paralogs: *GmLEC1-1, -2, -3*, and *-4*, with the first two more closely resembling Arabidopsis LEC1 in terms of their expression patterns. Chip-seq study using developing soybean embryos from various development stages including morphogenesis, transition and maturation stages suggested a functional conservation of LEC1 between Arabidopsis and soybean (Pelletier et al., 2017).

Overexpression of NF-YA1, 5, 6, and 9 can induce somatic embryogenesis from Arabidopsis seedlings, while overexpression of LEC1 and LIL leads to induced expression of NF-YA1, 5, and 9 (Mu et al., 2013). Arabidopsis plants overexpressing canola *BnLEC1* and *BnLIL* genes showed similar phenotypes as to Arabidopsis *AtLEC1* overexpression plants, suggesting the similarity of their functions (Mu et al., 2008). Overexpression of *AtLEC1* can induce embryonic transition in tobacco seedlings (Guo et al., 2013). In both zygotic and somatic embryos of the European larch, *LdLEC1* was highly expressed during early embryogenesis (Rupps et al., 2016). LEC1 was expressed during the induction phase of somatic embryogenesis and was linked to the embryogenic development in somatic cells in barrel clover (Orłowska et al., 2017). In callus tissue overexpressing *MtWOX9-1*, which exhibits enhanced somatic embryogenic potential, *MtNF-YB10*, a barrel clover homolog of Arabidopsis LEC1, displayed elevated and co-related expression with *MtWOX9-1* (Tvorogova et al., 2019). Rice LEC1 homologs *OsNF-YB9* and *OsNF-YB7* show different expression patterns with the former mainly expressed in endosperm, while the latter in embryo (Niu et al., 2021). However, overexpression of either *OsNF-YB9* or *OsNF-YB7* can restore Arabidopsis *lec1-1* mutants with more than 69.4% of seeds produced showing normal embryo morphology, suggesting a functional conservation of LEC1 between monocots and dicots. In addition, CRISPR/Cas9 knock-out of *OsNF-YB7* is fatal due to abnormal embryo development, providing further confirmation of its importance in rice embryogenesis. Though more research is needed to understand the organization of heterotrimeric complexes within the NF-Y subunits, it is evident that the NF-Y TF family plays a crucial role in plant embryogenesis.

### 3.6 Other TF families

In addition to the above-mentioned TFs, several others have been identified to have significant roles in plant embryogenesis. RWP-RK DOMAIN−CONTAINING PROTEIN (RKD) TFs are plant-specific transcription factors and five RKD genes were identified in Arabidopsis, namely *AtRKD1, AtRKD2, AtRKD3, AtRKD4*, and *AtRKD5* (Köszegi et al., 2011). Gene expression analysis shows that four of the Arabidopsis RKD genes *AtRKD1* to *AtRKD4* are mostly expressed in the reproductive tissues, with *AtRKD1* and *AtRKD2* mostly in egg cells, while *AtRKD4* in early embryos. Loss of function *rkd4* mutants fail to develop embryos properly and overexpression of RKD4 induces somatic embryogenesis (Waki et al., 2011). In addition, overexpression of *AtRKD1* and *AtRKD4* was able to induce somatic embryogenesis from suspensor cells (Radoeva et al., 2020). Maize RKD gene *Shohai1(Shai1)*, a close ortholog to *AtRKD5*, was shown to play an important role in embryo and endosperm development (Mimura et al., 2018). Loss of *Shai1* function leads to embryo defects, and the *shai1* mutant embryos can be partially rescued by overexpressing *Shai1* in the endosperm. *OsRKD3*, one of the rice RKD genes and the one that is closely related to *AtRKD4*, induces somatic embryogenesis in black rice (*Oryza sativa*) (Purwestri et al., 2023). A RKD homolog from Indonesian local pigmented rice (*Oryza sativa*), which is similar to *OsRKD*, is induced during the early stage of microspore embryogenesis (Nurbaiti et al., 2021). *CitRKD1* from satsuma mandarin was confirmed to be the candidate gene leading to somatic embryogenesis in citrus and transgenic sweet oranges with *CitRKD1* loss-of-function did not succeed in generating somatic embryos (Shimada et al., 2018). These studies show a conserved function of RKD genes in embryogenesis across various plant species.

GROWTH-REGULATING FACTORs (GRFs) are plant-specific transcription factors as well and multiple GRF genes have been identified in various species, with 9 GRFs in Arabidopsis, 13 in rice, 17 in maize and 26 in soybean (Omidbakhshfard et al., 2015). GRFs play important roles in various developmental processes including seed development with GRF1 or GRF5 overexpressing lines producing larger seeds in Arabidopsis (Van Daele et al., 2012). Overexpression of *AtGRF5* or respective GRF5 orthologs improved regeneration and transformation efficiency in both monocots such as maize and dicot species such as sugar beet (*Beta vulgaris ssp. Vulgaris*) including some recalcitrant sugar beet genotypes, canola, and soybean (Kong et al., 2020). Ectopic expression of *ZmGRF5-like1* and *ZmGRF5-like2* improved embryogenic callus growth as well as transformation efficiency in maize. GRFs and GRF-INTERACTING FACTORs (GIFs), a group of transcription cofactors, form a transcriptional complex to function and the complex is required for the development of meristematic and pluripotent cells (Lee et al., 2018). Overexpression of a wheat *TaGRF4* and *TaGIF1* chimeric protein improves embryogenesis and transformation efficiency in wheat, triticale and rice (Debernardi et al., 2020). The homologs of wheat *TaGRF4* and *TaGIF1* in citrus and grape (*Vitis vinifera*) were also used to generate a citrus GRF4-GIF1 chimera and a grape GRF4-GIF1 chimera. Both chimeras also increased transformation efficiency in citrus. Most recently, a new transformation system named GGB (GRF-GIF-BBM) utilizing *TaGRF4-GIF1* and *ZmBBM* was shown to increase the transformation efficiency in genome-edited maize genotypes with different genetic backgrounds (Chen et al., 2022b). It appears that the function of the GRF-GIF complex is conserved across monocot and dicot species, though the GRF-GIF complex mostly improves pluripotency (organogenesis), while it has a limited impact on totipotency (embryogenesis).

## 4 Transcription factor network during plant embryogenesis

Plant embryogenesis is a complex developmental process characterized by significant transformations, which require coordinated regulations of various genes, and transcription factors. It is evident that the key TFs we have emphasized in the preceding sections do not function in isolation during this process. Instead, they engage in active interactions with one another, as well as with their target genes, to establish complex transcriptional networks. These networks of TFs ensure the sequential and orderly progression of every stage of embryogenesis encompassing cellular reprogramming, patterning formation, and differentiation of meristematic tissues.

An *OsBBM1*-*OsYUC* module has been shown to play an important role in both zygotic embryogenesis and somatic embryogenesis in rice (Khanday et al., 2023). Paternal-genome-originated *OsBBM1* directly triggers maternal-genome-originated auxin biosynthesis gene *OsYUCCA* (*OsYUC*) to initiate zygotic embryo development; while under the culture conditions, exogenous auxin induces *OsBBM1*, which then activates endogenous *OsYUC* genes (*OsYUC6*, *OsYUC7* and *OsYUC9*) to promote somatic embryogenesis (Khanday et al., 2018; Khanday et al., 2023). A two-step model has also been proposed that cell totipotency is established through BBM-induced gene expression such as LEC1, LEC2, and FUS3, and then the induction of auxin biosynthesis is required for the maintenance of embryo identity and embryo development (Li et al., 2022). Auxin plays a fundamental role in pattern formation during embryogenesis. The above-mentioned master TFs such as BBM, LEC1, LEC2, and FUS3 have been reported to activate auxin biosynthesis genes such as YUCCA genes (YUCs) and there are complex feedback loops among these master TFs during embryogenesis (Kagaya et al., 2005; To et al., 2006; Yamamoto et al., 2010; Junker et al., 2012; Wójcikowska et al., 2013; Tian et al., 2020). A similar scenario of a coordinated TF regulatory network among these master TFs occurs in the context of Arabidopsis somatic embryogenesis. BBM and PLT2 activate the LAFL (LEC1-ABI3-FUS3-LEC2) network to induce somatic embryogenesis from Arabidopsis seedlings (Horstman et al., 2017b), while LEC2 promotes somatic embryogenesis through direct activation of WOX2 and WOX3 (Wang et al., 2020).

The regulatory network of MADS-domain TFs showed that AGL15 suppresses SEPALLATA3 (SEP3), but induces several important embryogenesis-related genes including FUS3, ABI3, as well as a gibberellin (GA) oxidase gene GA2ox6 while interacting with BBM and LEC1 (Wang et al., 2004; Zheng et al., 2009; Paul et al., 2022). Moreover, AGL18 induces AGL16, LEC1, PLETHORA2 (PLT2), and ABI4, while both AGL15 and AGL18 suppress GA3ox2, a gibberellin (GA) biosynthetic gene, to promote somatic embryogenesis (Paul et al., 2022). Earlier studies have shown that LEC2 and FUS3 negatively affect GA biosynthesis by repressing GA3ox2 expression, thus regulating the embryonic development (Curaba et al., 2004; Gazzarrini et al., 2004). The activation of auxin biosynthesis genes and the suppression of GA biosynthesis during embryogenesis further implies the importance of hormonal regulation during the process, however, delving into this aspect is beyond the scope of this review. Through the combination of ChIP-seq and RNA-seq studies, 283 genes were found to be induced and 472 genes were repressed in AGL15-overexpressed embryogenic cultures as compared to wild types (Joshi et al., 2022a). The relationship between AGL15 and other transcription factors, hormone genes, and genes involved in epigenetic modification, suggests a more complex network interaction in embryogenesis (Joshi et al., 2022b).

A BPC1 (Basic Pentacysteine 1)-FIS (Fertilization-independent Seed)-PRC2 (Polycomb Repressive Complex 2) network controls the spatiotemporal expression of FUS3 to coordinate embryo and endosperm growth (Wu et al., 2020). A number of embryogenic-related transcription factors have also been shown to be repressed by PRC 1 and 2 including WOX5, WOX8, AGL15, LEC1, LEC2, FUS3, ABI3, and BBM (Makarevich et al., 2006; Chen et al., 2010; Bouyer et al., 2011; Ikeuchi et al., 2015; Duarte-Aké et al., 2019). Polycomb Repressive Complexes (PRC 1 and 2) are one of the key components in epigenetic gene regulation and PRC2 leads to the trimethylation of histone H3 (H3K27me3) which will then activate PRC1-induced ubiquitination of histone H2A (H2Aub), processes that make chromatin transcriptionally inactive therefore repressing gene expression (Mozgova et al., 2015; Baile et al., 2022). As members of the B3 TF family VAL genes were initially identified in sugar signalling as High-level expression of sugar-inducible gene2 (HSI2/VAL1) and HSI2-LIKE1 (HSL1/VAL2) and expression of LEC1, LEC2, and FUS3 were induced in *hsi2 hsl1* mutants (Tsukagoshi et al., 2007). Chromatin immunoprecipitation shows that HSI2/VAL1 binds to AGL15 to repress its expression (Chen et al., 2018). Furthermore, increased evidence suggests that VAL genes interact, and recruit subunits of PRC for gene silencing as transcriptional repressors (Duarte-Aké et al., 2019; Yuan et al., 2021; Baile et al., 2022). However, as histone-combinational-binding effector proteins, GRFs recognize both histone phosphorylation and histone trimethylation at specific sites (H3K28ph and H3K27me3) to reverse the repression effects caused by these histone modifications (Zhao et al., 2018; Xu et al., 2022a). In addition, microRNAs also mediate transcriptional and post-transcriptional silencing of genes involved in plant development and microRNAs are mostly induced in non-embryogenic cultures than the embryogenic ones (Wu et al., 2015). MicroRNAs such as miR172 and miR1160, were shown to repress WUS and AP2/ERF TFs during the embryogenesis as well (Wu et al., 2015; Li et al., 2017; Nowak et al., 2022).

During early embryogenesis in rice, the spatiotemporal expression of multiple TFs including C2C2, homeobox, MADS, bHLH, and NAC showed preferential and divided expression patterns in distinct embryonic organs and domains (Itoh et al., 2016). These differences in the spatial expression of TFs suggested that the roles of TF family members in the initial patterning of the embryo and the arrangement of the embryonic organs were established at the early globular embryo stage (Itoh et al., 2016). The functions of WRKY2, HDG11 (HOMEODOMAIN GLABROUS 11), and WOX8/9 on suspensor development during early embryogenesis are conserved in plants and the activation of WOX8/9 is dependent on WRKY2 and HDG11 in addition to the feedback loop between WOX9 and HDG11 (Chen et al., 2021). RKD genes have been shown to induce a number of genes including AP2/ERFs, and MYBs during embryogenesis (Waki et al., 2011; Purwestri et al., 2023). Four TF classes, namely TEOSINTE BRANCHED1-CYCLOIDEA-PCF transcription factors (TCPs), Auxin response factors (ARFs), MYBs, and WOXs, were identified as playing central roles in the transcriptional regulation network during pattern formation of wheat zygotic embryos and a regulatory module involving LEC1-MYB118-ZHD5-LEC2-BBM was confirmed (Zhao et al., 2023). Briefly, LEC1, MYB118, and ZHD5 were induced first during the pro-embryo stage, followed by LEC2 to reach the peak at the transition stage, and then BBM at the mid-embryo stage. These spatial and sequential events show a coordinated TF regulatory network in charge of early embryogenesis.

**Figure 2:**
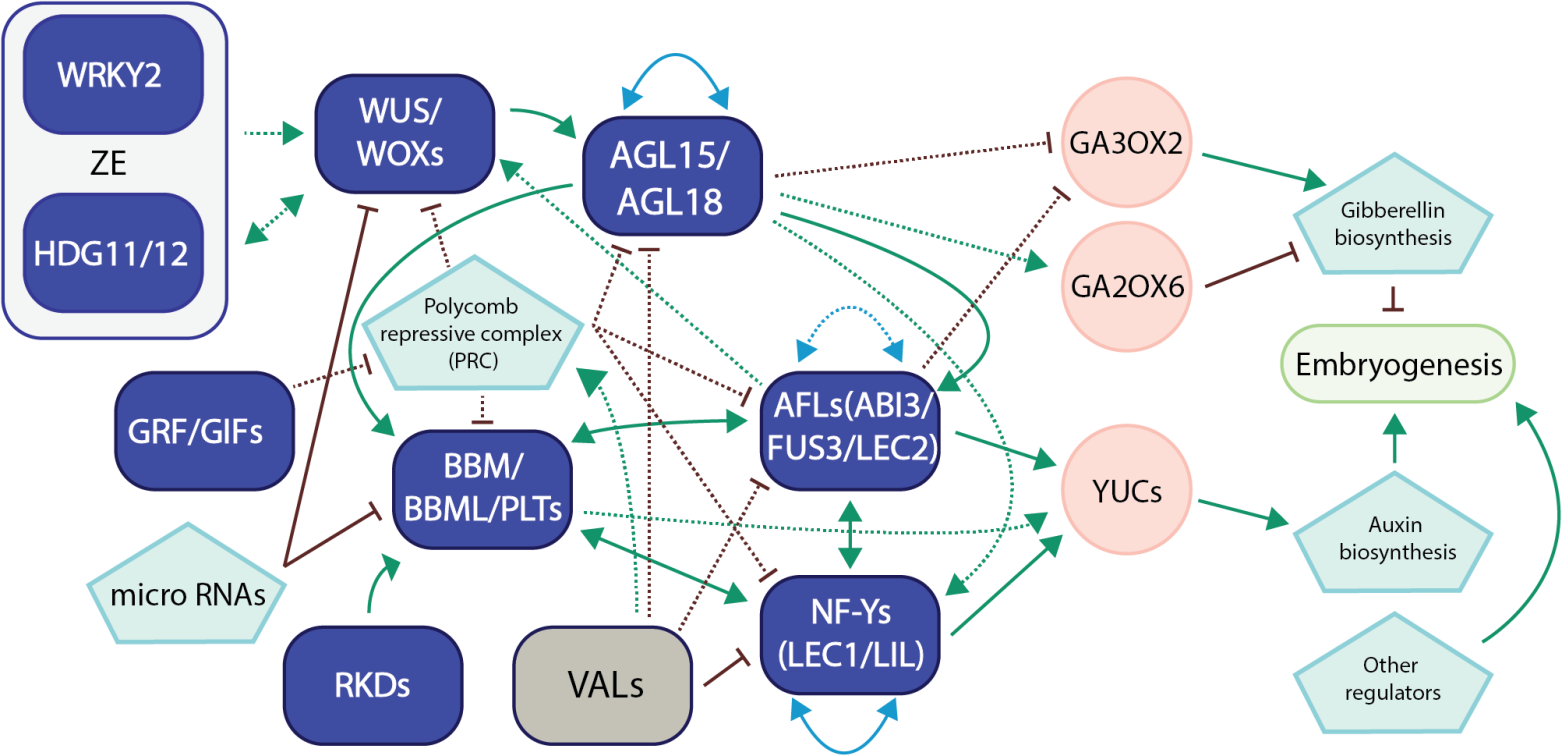
A representative model of transcription factor networks during plant embryogenesis. This model summarizes our recent research and the latest developments in the field regarding transcription factor networks in embryogenesis in Arabidopsis and other crop species (Horstman et al., 2017a; Pelletier et al., 2017; Ueda et al., 2017; Chen et al., 2018; Kong et al., 2020; Tian et al., 2020; Wang et al., 2020; Chen et al., 2022a; Kerstens et al., 2022; Paul et al., 2022; Khanday et al., 2023; Purwestri et al., 2023; Zhao et al., 2023). Transcription factors in purple usually promote embryogenesis, while TFs in gray suppress the process. For TF network interaction, solid lines refer to gene regulation, while dotted lines refer to protein-protein interaction or both. Green arrows refer to direct activation, while red bars refer to suppression. Blue lines with arrows on both ends refer to autoregulation/self-regulation or regulation between group members. Gray regions were used to show TF networks that have only been verified/proposed in zygotic embryogenesis (ZE). For the sake of clarity, we have included only the most significant networks and references.

A hierarchical transcriptional regulatory network for somatic embryogenesis was also identified through a combinational approach utilizing ATAC-seq, ChIP-seq, RNA-seq, and genetic transformation of immature Arabidopsis embryos (Wang et al., 2020). The sequential TF functions included bHLH and BES1 (BRI1-EMS-SUPPRESSOR1) TFs at the early stage of somatic embryogenesis, WRKY and CAMTA1 (calmodulin binding transcription activator 1) TFs from 0 to 8 hours after the initiation of the process, and then ARF, AP2, B3, and TCP (TEOSINTE BRANCHED1/CYCLOIDEA/PCF) TFs including BBM and LEC2 after 24 hours after the initiation. This sequential action of various TFs indicates a well-organized transcriptional regulatory network during somatic embryogenesis, underscoring the significance of transcription factor networks during plant embryogenesis. We have proposed a model for the network interactions involving these master transcription factors in plant embryogenesis, drawing upon recent advancements and our own research (**Figure 2**).

## 5 Future perspectives and conclusion

Cells undergo a series of intricated processes to develop into embryos with specific spatial organization, therefore the position of each cell within an embryo is critical for its function. Spatial-temporal single-cell transcriptomics can uncover gene expression of individual cells in various locations within an embryo throughout the course of embryogenesis at an unparalleled resolution. Therefore, spatiotemporal single-cell gene expression study during embryogenesis could be a valuable resource for functional analyses of transcription factors and a foundation for comparative studies of plant embryogenesis.

Overexpression of an individual transcription factor can lead to somatic embryogenesis in various plant species. Some of these master TFs include AP2 TF BBM/PLTs, Homeobox TF WUS/WOXs, B3 TF LEC2/FUS3, NF-Y TF LEC1, and RKD4 as described above. The combinational usage of two TFs such as BBM and WUS, or even more, such as the GGB (GRF-GIF-BBM) system, not only stimulates somatic embryogenesis in recalcitrant genotypes under hormone-free culture conditions but also dramatically speeds up the embryogenesis process. Could functional and mechanistic studies reveal additional master TFs? A better understanding of the functions and molecular mechanisms of these master TFs and a diverse selection of them will greatly improve somatic embryogenesis and increase transformation efficiency in a genotype-independent manner in crop species. This will drastically increase the applications and impacts of gene transformation and gene editing technologies for crop improvement through embryogenesis.

Over the past decade, much progress has been made in understanding the functions of TFs in plant embryogenesis and the molecular mechanisms related to these functions. TF regulatory networks involved in plant embryogenesis have gradually come to light, though much more research is needed to better understand the networks and their interactions during the process. The functional redundancy and divergence exhibited by TFs within the same family suggest a much more intricate regulatory process. How do the DNA-binding properties shared by TFs within the same family affect their target genes and the related regulatory networks is an intriguing question. The rapid technology development and applications such as ChIP-seq will provide powerful solutions to identify target genes of the TFs and the related TF regulatory networks during plant embryogenesis.

## Author contributions

HY: Conceptualization, Funding acquisition, Writing – original draft. SK: Funding acquisition, Writing – review & editing. AF: Funding acquisition, Writing – review & editing.
